# In Vitro Assessment of the Bioaccessibility of Zn, Ca, Mg, and Se from Various Types of Nuts

**DOI:** 10.3390/foods12244453

**Published:** 2023-12-12

**Authors:** Justyna Moskwa, Sylwia Katarzyna Naliwajko, Anna Puścion-Jakubik, Jolanta Soroczyńska, Katarzyna Socha, Wojciech Koch, Renata Markiewicz-Żukowska

**Affiliations:** 1 Department of Bromatology, Faculty of Pharmacy with Division of Laboratory Medicine, Medical University of Bialystok, Mickiewicza 2D, 15-222 Bialystok, Poland; sylwia.naliwajko@umb.edu.pl (S.K.N.); anna.puscion-jakubik@umb.edu.pl (A.P.-J.); jolanta.soroczynska@umb.edu.pl (J.S.); katarzyna.socha@umb.edu.pl (K.S.); renmar@poczta.onet.pl (R.M.-Ż.); 2 Department of Food and Nutrition, Medical University of Lublin, 4a Chodźki Str., 20-093 Lublin, Poland; kochw@interia.pl

**Keywords:** edible nuts, zinc, calcium, magnesium, selenium, in vitro bioaccessibility

## Abstract

The bioaccessibility of zinc (Zn), calcium (Ca), magnesium (Mg), and selenium (Se) from various nuts (Brazil nuts, walnuts, peanuts, almonds, cashews, pecans, hazelnuts, macadamia nuts, and pistachios) was assessed using a simulated two-phase model of enzymatic digestion in vitro. The levels of Zn, Mg, and Ca were determined by atomic absorption spectrometry, and Se was measured by inductively coupled plasma-mass spectrometry. All tested nuts were good sources of Mg, and most, except macadamia nuts, were also good sources of Zn (the standard portion covers over 15% of NRV–R (UE) 1924/2006). Brazil nuts had the highest Se content. Almonds and Brazil nuts were rich in Ca. Se demonstrated the highest bioaccessibility from nuts (27.7% to 70.65%), whereas Ca exhibited the lowest bioaccessibility (below 9%). Pistachios had the highest Zn bioavailability, while cashews excelled in Mg bioaccessibility. Macadamia and pistachios were top for Ca bioaccessibility, and Brazil nuts for Se. Bioaccessibility is positively correlated with fat (for Zn: r = 0.23), carbohydrates (for Mg: 0.44; for Ca: 0.35), and sugar content (for Zn: r = 0.36; for Mg: 0.46; for Ca: 0.40).

## 1. Introduction

Edible nuts, renowned for their abundant nutritional content and health-enhancing qualities, are a vital component of a balanced human diet. Nuts typically contain macronutrients, with a high proportion of unsaturated fats, dietary fiber, vitamins, phenolic compounds, and minerals [1,2,3]. Several research studies have reported that nuts can help reduce the risk of cardiovascular diseases by lowering total cholesterol levels, particularly the atherogenic LDL fraction, as well as decreasing triacylglycerols in the bloodstream. This may also depend on the mineral content of the nuts. Jamilian et al. conducted a randomized study among 70 women (18–40 years old) diagnosed with polycystic ovary syndrome; participants took 200 µg of selenium for 8 weeks. The results demonstrated significant reductions in serum triglycerides (TG), very-low-density lipoprotein (VLDL-C), and insulin compared to placebo [4]. In a study by Hamedifard et al., it was shown that the administration of Zn and Mg (150 mg and 250 mg, respectively) for 12 weeks to patients with coronary artery disease and type 2 diabetes resulted in a decrease in glucose and insulin levels and an increase in HDL cholesterol [5]. Antioxidants, primarily phytosterols found in nuts, reduce the concentration of atherogenic forms of oxLDL-cholesterol and lower the risk of DNA damage [6,7,8]. The studies have indicated that nut consumption can have a positive effect on inflammatory markers by lowering their levels [9]. Dietary guidelines for diabetes recommend patterns that incorporate nuts due to their association with several beneficial health effects. Studies indicate that the minerals and bioactive compounds found in nuts can help regulate glycemic and insulin levels and contribute to obesity reduction [10,11]. Some studies have demonstrated that nuts and edible seeds possess anti-mutagenic, anti-microbial, and anti-inflammatory potential [12,13,14]. 

Numerous studies in the literature have assessed the levels of bioactive compounds in nuts [15,16,17]. Nevertheless, comprehension of the complete content of essential substances in food is insufficient to ascertain the beneficial or detrimental effects of its consumption on human health. Therefore, it becomes imperative to acquire knowledge regarding the quantities of these substances that enter the systemic circulation following ingestion and become accessible for the induction of biological activity—a concept known as bioavailability [18]. Determining the total content of substances in food products should result from knowing the level of these substances released during digestion and absorption in the gastrointestinal tract [19]. From a nutritional perspective, the issue of macro- and micronutrient bioavailability is crucial, both in terms of dietary intake and supplement utilization. The bioavailability of a mineral or trace element is defined as the fraction of an ingested nutrient that undergoes absorption and subsequently becomes essential for physiological functions [20]. However, it is worth noting that factors such as age, gender, health status, or the physiological state of the gastrointestinal tract, as well as the type of diet, are important factors that significantly influence bioavailability and can only be thoroughly assessed through clinical studies. In vitro methods designed to mimic gastrointestinal processes depend on various gastrointestinal parameters, including pH levels, enzymatic activities, temperature, and mixing dynamics [21]. Despite the inherent limitations of these methods, their notable advantages lie in their simplicity, cost-effectiveness, and ethical neutrality. They do not require specialized and expensive equipment and are thus frequently employed for preliminary estimation of element bioaccessibility in food products [22]. Other authors have described the bioaccessibility of some minerals in various nuts and seeds [17,23], but in our study, for the first time, we comprehensively examined the bioaccessibility and mineral content in a varied group of nuts.

Our research aimed to evaluate the bioaccessibility of elements (Zn, Mg, Ca, and Se) in Brazil nuts, walnuts, peanuts, almonds, cashew nuts, pecans, hazelnuts, macadamia nuts, and pistachios (n = 90) purchased at local supermarkets. We employed a simulated two-phase in vitro model, mimicking the processes in the stomach and the small intestine. This model involved the utilization of gastric and intestinal enzymes, as well as cellulose membranes, followed by quantification of the released contents of Zn, Mg, Ca, and Se subsequent to enzymatic digestion.

## 2. Materials and Methods

### 2.1. Reagents

Analytical-grade chemicals and ultra-pure water (Simplicity 185, Millipore, Bedford, MA, USA) were used in all procedures. Standard solutions of Zn, Ca, Mg, and Se were prepared from stock solutions of 1000 mg/L (Merck, Darmstadt, Germany). For the acid digestion procedure, spectrally concentrated nitric acid at 69% concentration was utilized (69% HNO_3_, Tracepur, Merck, Darmstadt, Germany). Lanthanum (III) chloride hydrate (Sigma Aldrich, St. Louis, MO, USA) served as a modifying reagent for the determination of Ca and Mg. The digestive enzymes used, namely pepsin from porcine gastric mucosa (77160-100g), bile salts (48305-50g-F), and pancreatin from porcine pancreas (P1750-100G), along with dialysis tubing cellulose membrane (D9777-100FT), were all procured from Sigma Aldrich (St. Louis, MO, USA). Hydrochloric acid, with a concentration of 36.5 to 38%, which is suitable for metal trace analysis (7647-01-0), was sourced from J.T. Baker Instra-Analyzed (Phillipsburg, NJ, USA).

### 2.2. Sample Collection

We conducted an investigation on a total of 90 samples, composed of a variety of nuts, including Brazil nuts, walnuts, peanuts, almonds, cashew nuts, pecans, hazelnuts, macadamia nuts, and pistachios. The samples, selected to be representative of the broader Polish market, were obtained from local grocery stores in north-eastern Poland, with 10 samples collected for each nut type, each from a different producer. The countries of origin of the tested samples were: Africa (n = 2), Argentina (n = 4), Bolivia (n = 8), Brazil (n = 3), China (n = 1), Egypt (n = 1), Georgia (n = 3), Spain (n = 8), India (n = 1), Iran (n = 4), Kenya (n = 6), Mexico (n = 1), Nigeria (n = 2), Poland (n = 12), South Africa (n = 2), Turkey (n = 5), USA (n = 14), and Vietnam (n = 3). Country-of-origin details were unavailable for 10 products. The content of nutrients (protein, fat, saturated fatty acids, carbohydrates, and sugars) was derived from the nutritional information provided on the product labels. The nut samples were ground using a mortar, subsequently sealed in polyethylene bags, and stored at a temperature of 4 °C until the time of analysis.

### 2.3. The In Vitro Digestion Model

**Figure 1:**
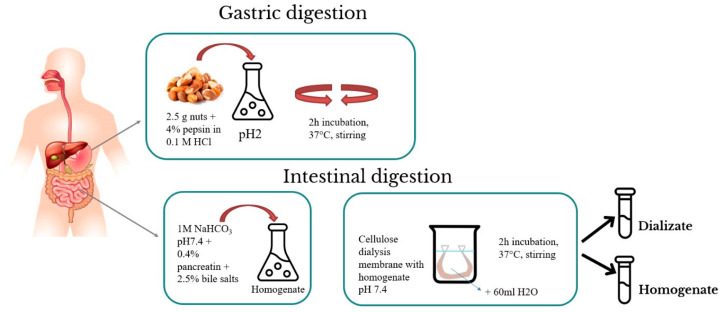
In vitro digestion procedure.

The methodology for investigating the in vitro bioaccessibility of the elements was adapted with some modifications from procedures by Miller et al. [24] and Moreda-Piñeiro et al. [17]. The method additionally used cellulose dialysis tubing to approximate more natural conditions (Figure 1). The samples (2.5 g) were weighed and then homogenized with 15 mL of a 4% pepsin solution prepared in 0.1 M hydrochloric acid (HCl). These mixtures (pH = 2) were incubated at 37 °C for 2 h in a thermostatic shaking water bath. In the subsequent step, the required amount of 1M sodium bicarbonate (NaHCO_3_) was added to the solution to achieve a pH similar to that of the intestinal tract (pH = 7.5), and the quantity added was recorded. After achieving the appropriate pH value in each system, we added 5 mL of 0.4% pancreatin and 2.5% bile salts in 0.1 mol/dm^3^ NaHCO_3_ to each sample. Prior to placing the samples in cellulose dialysis tubes (12 kDa MWCO), their pH (7.5) was obtained, and enzymes were added to the samples. The tubes were properly prepared (treated with 0.1 M HCl for 12 h and washed out). Properly protected tubes were transferred to containers with 60 mL of ultrapure water. In the next step, they were placed in a water bath and incubated with shaking for 2 h at 37 °C. After the digestion process, two types of samples were obtained (dialysates and residues from the tube). Samples were stored at −20 °C until analysis. Control samples were subjected to the same process. 

### 2.4. Microwave-Assisted Acid Digestion

Weighed raw samples, approximately 0.3 g (±0.001 g), were placed in mineralization polytetrafluoroethylene vessels. Subsequently, 4 mL of spectrally pure, concentrated (69%) HNO_3_ was added (Tracepur, Merck, Darmstadt, Germany). The microwave digestion process was performed in a closed-loop system (Berghof, Speedwave, Eningen, Germany), involving five steps, as outlined in Table 1. 

The dialysate and residual fractions underwent a microwave-assisted acid digestion procedure as well. In this instance, we weighed 4 g of dialysate and 1 g of residual fraction from each sample, to which we added 4 mL of spectrally pure, concentrated HNO_3_ (69%). Subsequently, the next microwave digestion process was conducted. Following mineralization, the samples were diluted appropriately with ultrapure water. 

### 2.5. Analytical Determination of Zn, Mg, and Ca by Atomic Absorption Spectrometry (AAS)

The concentrations of Zn, Mg, and Ca were determined using AAS (Z-2000 Tandem Flame/Furnace AA Spectrophotometer, Hitachi, Japan) with flame atomization in an acetylene-air flame. Zn was measured at a wavelength of 213.9 nm, Mg at 285.2 nm, and Ca at 422.7 nm, all with Zeeman background correction. To determine Ca and Mg contents, a 1% solution of lanthanum (III) chloride hydrate (Sigma Aldrich, St. Louis, MO, USA) was used as the phosphate masking reagent. The detection limits (LOD) for Zn, Mg, and Ca were: 0.017 mg/kg, 0.01 mg/kg, and 0.17 mg/kg, respectively. 

#### Quality Control

Quality control was performed using certified reference materials (CRMs) for Simulated Diet D (Livsmedelsverket, National Food Agency, Uppsala, Sweden). The contents of individual elements in the CRMs were determined. The results of the quality control for analytical methods demonstrated a precision of 3.1%, 2.7%, 2.8%, and 2.4% for the determination of Zn, Ca, Mg, and Se, respectively. The percent recoveries for the determination of Zn, Ca, Mg, and Se were 99.9%, 101.2%, 99.6%, and 98.4%, respectively. The declared concentration in the CRMs for Zn, Ca, Mg, and Se was as follows: 37.2 ± 3.1 mg/kg, 510 ± 37 mg/kg, 676 ± 65 mg/kg, and 0.214 ± 0.014 µg/kg, respectively.

### 2.6. Analytical Determination of Se by Inductively Coupled Plasma-Mass Spectrometry (ICP-MS)

The concentration of Se was determined using inductively coupled plasma-mass spectrometry (ICP-MS, NexION 300D, PerkinElmer, Waltham, MA, USA) with kinetic energy discrimination (KED). High-purity helium (He, 99.999%) served as the transport gas to carry the sample aerosol from the ablation chamber to the ICP-MS instrument. Polyatomic interferences were corrected through collisions and kinetic energy discrimination. Concentrations were calculated based on calibration curves. The results were reported in counts per second (cps). A standard calibration curve was established using a Se solution with a concentration of 10 µg/L (PerkinElmer, Boston, MA, USA). The ICP-MS conditions were as follows: mass values: 76.9199, 77.9173, and 81.9167 amu; dwell time per amu: 50 ms; integration time: 1000 ms; and dual detector calibration mode. The limit of detection (LOD) value for Se determination was 0.01 µg/L.

### 2.7. Microwave-Assisted Acid Digestion

The bioaccessibility ratios of the elements (Zn, Mg, Ca, and Se), expressed as a percentage, were calculated using the following equation:

$$B\%=\left(\frac{D+Dr\;}{T+D}\right)\times 100\%$$

where

B%—% of the bioaccessibility (relative bioaccessibility) of tested elements,
D—the amount of the mineral (mg) in the dialysate,
Dr—the amount of the mineral (mg) corresponding to the equilibrium of concentrations on both sides of the cellulose membrane inside the dialysis tube,
T—the amount of the mineral (mg) present in the digest of the dialysis tube residue.

Dr was calculated using the following equation:

$$Dr=\frac{\left(Cd-Ck\right)\times Vt}{Vd}$$

where

Cd—the concentration of the mineral in the dialysate solution (g/mL),
Ck—the concentration of the mineral in the control sample (g/mL),
Vt—the volume of the dialysis tube (mL),
Vd—the volume of dialysate (mL).

### 2.8. Nuts as Sources of Dietary Minerals 

The recommended daily allowances (RDAs) [25] and nutrient reference values (NRVs), along with the Index of Nutritional Quality (INQ), have been used to assess the potential of nuts in supplementing dietary deficiencies of Zn, Mg, Ca, and Se. According to the European Commission, a food product is considered a valuable source of a mineral if its standard portion covers over 15% of the NRV for that particular mineral. The NRVs for the minerals discussed in our study are set at 10 mg for Zn, 375 mg for Mg, 800 mg for Ca, and 55 µg for Se (Regulation (EU) no. 1169/2011 of the European Parliament and of the Council; European Commission (EC) Regulation (Ec) No. 1924/2006 of the European Parliament and of the Council on Nutrition and Health Claims Made on Foods) [26,27]. To estimate the RDA percentage, we adopted a serving weight of 42 g, an amount commonly utilized in dietary intervention studies [6]. The INQ, which represents a ratio of the nutrient-to-calorie content of foods [28], can be calculated according to the following formula:

$$INQ=\frac{A\times B}{C\times D},$$

where

A—content of the tested element in 100 g of the study product
B—the standard of energy demand (women: 2300 kcal; men: 3150 kcal);
C—energy from 100 g of the product (Brazil nuts: 683 kcal; walnuts: 684 kcal; peanuts: 606 kcal; almonds: 616 kcal; cashews: 587 kcal; pecans: 708 kcal; hazelnuts: 692 kcal; macadamia nuts: 735; pistachio nuts: 591 kcal);
D—the requirement for the tested elements depending on age and gender (women: Ca—1000 mg, Mg—320 mg, Se—55 µg, and Zn—8 mg; men: Ca—1000 mg, Mg—420 mg, Se—55 µg, and Zn—11 mg).

An INQ value > 1 indicates that the product is a valuable source of nutrients and can be used to supplement dietary deficiencies.

### 2.9. Statistical Analysis

To analyze the data, we used Statistica software v. 13 (Tibco, Palo-Alto, CA, USA). Average value (A.V.) with standard deviation (SD), minimum (Min.), maximum (Max.), median (Med.), and first and third quartiles (Q1–Q3) were calculated in Excel (Office, Microsoft). The normality of the data distribution was assessed using the Shapiro–Wilk test. The values of the mineral content and bioaccessibility between nut types were examined using Kruskal–Wallis analysis of variance (ANOVA) with post hoc analysis and the Mann–Whitney U test. The relationships between the contents of the examined elements were evaluated using Spearman’s rank correlation. Feature similarity analysis was conducted using the Principal Component Analysis (PCA) method. Differences were considered statistically significant when the *p*-value was < 0.05.

## 3. Results and Discussion

### 3.1. The Concentration of Zn, Mg, Ca, and Se in Nuts

The results obtained for the total content of Zn, Mg, Ca, and Se in nine different types of nuts are given in Table 1. The content of these minerals varied considerably among the types of nut samples, as confirmed by the Kruskal–Wallis ANOVA test. The median Zn content in the studied nuts ranged from 16.2 mg/kg in macadamia nuts to 76.6 mg/kg in cashew nuts. Cashew nuts, Brazil nuts, and pecans were characterized by the highest content of Zn among the tested nuts. Moreda-Piñeiro et al. [17] reported slightly higher Zn concentrations in their study: almonds ranging from 30.3 to 38.8 mg/kg, cashew nuts with 46.6 mg/kg, walnuts with 29.9 mg/kg, peanuts with 34.4 mg/kg, and macadamia nuts with 12.5 mg/kg. In our study, the highest concentration of Mg was found in cashew nuts (5206.3 mg/kg) and peanuts (4849.3 mg/kg) (Table 1). Other types of nuts also contained considerable amounts of Mg. The highest concentrations of Ca were found in almonds and Brazil nuts, with medians of 4899.6 mg/kg and 3927.7 mg/kg, respectively, significantly higher than the other nuts. Suliburska and Krejpcio [29] reported similarly high concentrations of Mg and Ca in Brazil nuts, cashews, and hazelnuts. 

The measurements of Se content revealed that Brazil nuts definitely had the highest concentration of this element, ranging from 804.1 to 8982.3 µg/kg, i.e., significantly higher compared to Se content in the other tested nuts. The obtained results are consistent with those reported by many other authors [15,30,31]. The apparent differences in the levels of the macronutrients and micronutrients in the tested nuts may depend on a number of factors, such as plant origin, location and method of cultivation, soil composition, time of harvest, storage conditions, the possibility of contamination, and others [29,32].

### 3.2. Nuts as Sources of Zn, Mg, Ca, and Se

**Figure 2:**
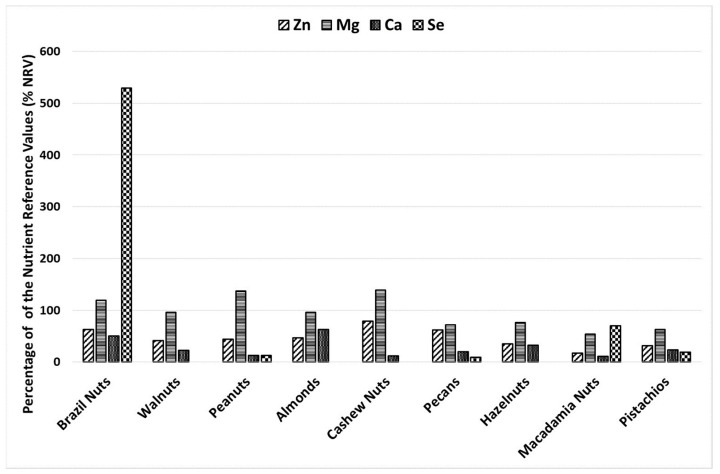
Coverage (in %) of the nutrient reference value (NRV) for Zn, Mg, Ca, and Se for an adult through the consumption of 100 g of nuts.

Edible nuts can be considered a natural source of macro- and micro-elements according to the recommended daily allowance (RDA), the value estimation of the Index of Nutritional Quality (INQ) (Table 2), and the nutrient reference value (NRV) (Figure 2). All the tested nuts, except for macadamia nuts, cover over 15% of the NRV and are sources of Zn (according to EU regulation). These products can supplement Zn deficiencies in the diet (INQ value: 1.5–3.9) and provide between 12% (pistachios) and 41% (cashew nuts) of the RDA for Zn in a recommended portion (42 g). All the tested nuts are good sources of Mg, and the consumption of a recommended portion fulfills from 20% (macadamia nuts) to 68–69% (peanuts and cashews) of the RDA. INQ values for Mg are above 1 (ranging from 2.0 to 6.7) (Table 2). All the tested nuts cover over 15% of the NRV and are a source of Mg (according to EU regulation). 

In general, most of the tested nuts are not good sources of Ca. The consumption of 42 g of walnuts, peanuts, cashew nuts, pecans, macadamia nuts, and pistachios per day fulfills only 4–8% of the RDA for Ca. However, almonds and Brazil nuts had an INQ >1 and fulfilled the RDA by more than 15% (21% and 17%, respectively). The NRV (800 mg/d) is lower than the RDA (1000–1200 mg/d), and only peanuts, cashews, and macadamia nuts cannot be regarded as a source of Ca. Almonds and Brazil nuts fulfill the NRV at 63.1 and 50.3%, respectively. Only Brazil and macadamia nuts can be regarded as good sources of Se. Other types of nuts contain very small or undetected amounts of Se (Table 2). The consumption of the recommended daily portion (42 g) of macadamia nuts covers 30% of the RDA, and the INQ is 2.2–3.0 for females and males. Brazil nuts contain the highest amounts of Se; a recommended daily portion provides 222% of the RDA, and 100 g covers more than 500% of the NRV. It should be noted here that the consumption of Brazil nuts in such significant amounts can lead to Se toxicity. Among those tested, consumption ranging from 6.1 g to 68.4 g (median 32.4 g) of Brazil nuts (equivalent to 1.5 to 17 pieces of nuts) covers the NRV by 100%. 

RDA, NRV, and INQ are based on recommended intakes and include the average bioaccessibility of various elements. In this paper, the bioaccessibility of elements was determined using an enzymatic model of in vitro digestion.

### 3.3. Bioaccessibility of Zn, Mg, Ca, and Se in Nuts

Table 3 presents the in vitro bioaccessibility of Zn, Mg, Ca, and Se in nuts, along with correlations (shown in Table 4) between the bioaccessibility of the tested minerals and the content of basic nutrients. It was found that the highest bioaccessibility percentages for Zn were recorded for pistachios (48.1%) and macadamia nuts (39.4%). The other authors reported lower bioaccessibility percentages for Zn in pistachios (9.6%) and macadamia nuts (22.5%) [17]. Arpadjan et al. [23] investigated the in vitro bioaccessibility of Zn and other elements (Fe, Cu, Mn, Cd, and Pb) in walnuts and hazelnuts. The bioaccessibility of Zn for hazelnuts was higher (59–62%) than in our study (9.7%). The bioaccessibility of the elements found in walnuts decreased markedly during intestinal digestion, dropping from 27−29% in the gastric tract to 14−16%, which aligns with our results (10.7%) (Table 3). The other study showed lower bioaccessibility of Zn in hazelnuts (1.9%), similar in walnuts (11.4%), and higher in Brazil and cashew nuts (20.6% and 52.4%, respectively) compared to the nuts examined in our experiment [29]. When analyzing the bioaccessibility of Mg and Ca, we obtained reasonably good agreement with the results reported by other authors [17]. The highest bioaccessibility of Mg was observed for cashew (27.0%) and macadamia nuts (24.0%). Ca showed relatively low bioaccessibility in the tested nuts, which is consistent with the findings by other authors [17]. The bioaccessibility of Ca from foods may depend on several factors, including solubility, concentration in the gastrointestinal tract, or the time it takes to digest. Only the ionized form of calcium can be absorbed. This is influenced by the correspondingly low pH in the stomach [33]. The stage of dialysis across the membrane in the simulated in vitro digestion model may be the reason for the low bioaccessibility of Ca in this experiment. Ca may have been enzymatically released from the sample but was unable to cross the dialysis membrane [17].

After simulated gastrointestinal digestion, the bioaccessibility percentage of Se was the highest for Brazil nuts (71%) (Table 3). Comparable results were obtained in the study by Gomes da Silva et al. [34], where the bioaccessibility percentage was 74%. The other authors reported a lower percentage [17]. However, it must be mentioned that the results may be attributed to the method’s setting, reagents used, experimental conditions, and possible sample contamination, which may have significantly influenced the outcome of the experiment. Thomson et al. [35] conducted a randomized controlled trial on Se bioaccessibility with adults who consumed Brazil nuts for 12 weeks. After that time, plasma Se levels increased by 64.2%. In general, the bioaccessibility of Se is highly dependent on its chemical form (organic or inorganic) and place of origin [36]. Se in Brazil nuts is found mainly in the organic form—selenomethionine [30]. The high content of Se in Brazil nuts is attributed to the presence of proteins composed of sulfur-containing amino acids. As a result, selenomethionine may not specifically replace methionine [37]. 

The bioaccessibility of minerals in all nuts showed a positive correlation with fat content (for Zn: r = 0.23), sugars (for Zn: r = 0.36, for Mg: 0.46, for Ca: 0.40), and carbohydrates (for Mg: 0.44, for Ca: 0.35) (Table 4). The bioaccessibility of Se was not included in the correlation analysis due to the substantial variance in results.

So far, research on the impact of basic nutrients on the bioaccessibility of selected ingredients has focused on substances with antioxidant properties. It has been shown, among others, that there is an inverse relationship between the total fat content of nuts and the bioaccessibility of hydrophilic phenolic compounds [38].

### 3.4. Principal Component Analysis

The next statistical analysis performed was principal component analysis (PCA), considering parameters such as the bioaccessibility of Zn, Mg, Ca, and Se, as well as the content of fat, saturated fat, carbohydrates, sugars, fiber, protein, salt, and energy value (Table S1). The analysis revealed that three factors with eigenvalues greater than 1 accounted for 84.3% of the variance. The first principal component, explaining 45.0% of the total variance, was predominantly influenced by the content of saturated fats, carbohydrates, and sugars. The second component, explaining 30.0% of the variance, encompassed variables such as the bioaccessibility of Zn, Mg, Ca, fat content, protein, and energy value. The third component, explaining 9.3% of the variance, included the bioaccessibility of Se along with the content of saturated fat, fiber, and salt. These results suggest a relationship between the bioaccessibility of minerals and the content of essential nutrients. Research conducted by Moreda-Piñeiro et al. (2016) also indicates that the bioaccessibility of minerals (Ca, Mg, but also Cu, Mn, Ni, and P) is mainly related to the fat and carbohydrate contents [17].

### 3.5. Future Perspectives and Limitations of the Study

Studies related to bioaccessibility assessment are based on isolated nutrients or simple systems. It should be emphasized that the bioaccessibility of ingredients is inextricably linked to the food matrix in which they occur, so future research models should reproduce not only the effect of enzymes but also the composition of the test product (including the content of protein, fats, and carbohydrates, among others) to best reproduce real-world conditions.

The limitation of this study was that it used another method than the recommended standardized INFOGEST 2.0 static in vitro digestion method, but we will consider its use in future studies.

## 4. Conclusions

The content of minerals (Zn, Mg, Ca, and Se) varies significantly depending on the nut variety. It was found that all the tested nuts (Brazil nuts, walnuts, peanuts, almonds, cashew nuts, pecans, hazelnuts, macadamia nuts, and pistachios) can be considered good sources of Mg. Most of the studied nuts, with the exception of macadamia nuts, can also serve as sources of Zn. Brazil nuts are the best source of Se, while almonds and Brazil nuts are the best sources of Ca. Nevertheless, it should be noted that despite the high content of minerals in nuts, their bioaccessibility, which is affected by the food matrix, varies significantly. Pistachios showed the highest bioaccessibility of Zn, cashews of Mg, macadamia nuts and pistachios of Ca, and Brazil nuts of Se. Se was found to be the element best absorbed from nuts (27.7–70.65%), while Ca exhibited the lowest bioaccessibility (<9%). The bioaccessibility of minerals was positively correlated with fats, carbohydrates, and sugars. It can be concluded that incorporating these nuts into one’s diet can improve the quality of nutrition. The research conducted contributes new knowledge that holds significance from both health and dietary perspectives.

## Figures and Tables

**Table 1:** Mineral concentrations of Zn, Mg, Ca, and Se in different types of nuts (Brazil nuts, walnuts, peanuts, almonds, cashew nuts, pecans, hazelnuts, macadamia nuts, and pistachios).

Types of Nuts	Zn [mg/kg]		Mg [mg/kg]	
	Average ± SD Min–Max	Median Q1–Q3	Average ± SD Min–Max	Median Q1–Q3
Brazil Nuts (a)	63.4 ± 6.2 (56.4–72.7)	61.8 (57.1–68.4)	4467.1 ± 1362.2 (3191.5–8060.7)	3994.4 (3857.2–4674.9)
Walnuts (b)	41.6 ± 3.9 (35.8–46.7)	43.2 (37.8–45.0)	3597.0 ± 1116.7 (2340.3–6167.8)	3189.6 (2854.2–4156.2)
Peanuts (c)	43.8 ± 6.4 (36.1–57.2)	41.9 (40.3–43.2)	5151.5 ± 772.0 (4204.7–6119.5)	4849.3 (4601.9–5950.2)
Almonds (d)	46.9 ± 6.9 (34.5–59.9)	47.5 (46.6–49.4)	3589.9 ± 765.9 (2240.5–4865.3)	3578.2 (3026.4–3918.3)
Cashew Nuts (e)	78.7 ± 5.7 (72.7–89.5)	76.6 (75.3–81.5)	5221.8 ± 933.5 (3561.7–6567.6)	5206.3 (4858.3–5903.5)
Pecans (f)	62.0 ± 10.2 (45.4–78.8)	60.3 (55.9–69.3)	2709.5 ± 687.1 (1595.5–3788.8)	2846.7 (2197.0–3112.5)
Hazelnuts (g)	35.1 ± 3.4 (31.7–42.4)	34.9 (31.9–36.6)	2874.2 ± 977.3 (1765.6–5006.9)	2592.2 (2182.8–3667.8)
Macadamia Nuts (h)	17.6 ± 3.6 (14.3–26.2)	16.2 (15.6–17.9)	2033.0 ± 470.4 (1505.1–3005.1)	1872.0 (1778.7–2068.5)
Pistachios (i)	31.7 ± 6.0 (23.4–42.7)	30.5 (29.8–34.7)	2355.7 ± 556.8 (1618.3–3042.6)	2415.8 (1805.4–2864.6)
Significant differences	*p* < 0.05: a/b, a/c/, a/d, a/e, a/g, a/h, a/i, b/d, b/e, b/f, b/g, b/h, b/i, c/e, c/f, c/g, c/g, c/h, c/i, d/e, d/f, d/g, d/h, d/i, e/f, e/g, e/g, e/h, e/i, f/g, f/h, f/i, g/h, h/i.		*p* < 0.05: a/b, a/c, a/e, a/f, a/g, a/i, a/h, b/c, b/e, b/h, b/i, c/d, c/f, c/g, c/h, c/i, d/e, d/f, d/h, d/i, e/f, e/g, e/h, e/i, f/h, g/h, h/i.	
	**Ca [mg/kg]**		**Se [µg/kg]**	
Brazil Nuts (a)	4020.8 ± 881.8 (2992.0–5381.5)	3927.7 (3189.7–4839.1)	2911.8 ± 2641.4 (804.1–8982.3)	1697.3 (1305.7–4697.6)
Walnuts (b)	1784.7 ± 493.8 (1130.7–2506.3)	1748.0 (1445.3–2229.9)	--	--
Peanuts (c)	1011.2 ± 265.8 (651.9–1417.1)	1085.6 (768.5–1163.9)	71.3 ± 48.2 (7.3–156.0)	60.1 (41.7–107.0)
Almonds (d)	5048.1 ± 463.0 (4514.3–5905.2)	4899.6 (4744.5–5278.1)	--	--
Cashew Nuts (e)	938.7 ± 440.3 (397.9–1668.2)	883.7 (605.8–1131.6)	257.9 ± 107.6 (124.7–424.1)	244.6 (157.3–356.5)
Pecans (f)	1563.6 ± 243.2 (1230.4–2115.1)	1521.1 (1455.1–1564.1)	65.9 ± 47.3 (23.8 –170.9)	50.9 (35.9–79.4)
Hazelnuts (g)	2575.8 ± 519.6 (1801.8–3577.9)	2637.5 (2094.5–2824.1)	--	--
Macadamia Nuts (h)	913.5 ± 321.3 (113.7–1264.8)	938.8 (844.6–1097.1)	387.3 ± 158.8 (177.1–623.5)	364.0 (245.3–560.6)
Pistachios (i)	1896.1 ± 486.2 (1238.8–2928.9)	1848.3 (1581.6–1968.8)	103.5 ± 47.4 (49.6–217.5)	97.7 (81.2–111.2)
Significant differences	*p* < 0.05: a/b, a/c, a/d, a/e, a/f, a/g, a/h, a/i, b/c, b/d, b/e, b/g, b/h, c/d, c/f, c/g, c/i, d/e, d/f, e/f, e/g, e/i, f/g, f/h, f/i, g/h, g/i, h/i		*p* < 0.05: a/c, a/e, a/f, a/h, a/i, c/e, c/h, e/f, e/i, f/h, f/i, h/i	

--—not detected.

**Table 2:** Percentage of recommended dietary allowance (RDA) for Zn, Mg, Ca, and Se in relation to the consumption of the recommended daily portion of nuts (42 g) and the value of the Index of Nutritional Quality (INQ).

**Types of Nuts**	**RDA (%)**							
	**Zn**		**Mg**		**Ca**		**Se**	
	**Male**	**Female**	**Male**	**Female**	**Male and Female**			
Brazil Nuts (a)	24	33	45	59	17		222	
Walnuts (b)	16	22	36	47	7		0	
Peanuts (c)	17	23	52	68	4		5	
Almonds (d)	18	25	36	47	21		0	
Cashew Nuts (e)	30	41	52	69	4		0	
Pecans (f)	24	33	27	36	7		4	
Hazelnuts (g)	13	18	29	38	11		0	
Macadamia Nuts (h)	7	9	20	27	4		30	
Pistachios (i)	12	17	24	31	8		8	
**Types of Nuts**	**INQ**							
	**Zn**		**Mg**		**Ca**		**Se**	
	**Male**	**Female**	**Male**	**Female**	**Male**	**Female**	**Male**	**Female**
Brazil Nuts (a)	2.7	2.7	4.9	4.7	1.9	1.4	24.4	17.8
Walnuts (b)	1.7	1.7	3.9	3.8	0.8	0.6	--	--
Peanuts (c)	2.1	2.1	6.4	6.1	0.5	0.4	0.7	0.5
Almonds (d)	2.2	2.2	4.4	4.2	2.6	1.9	--	--
Cashew Nuts (e)	3.8	3.9	6.7	6.4	0.5	0.4	--	--
Pecans (f)	2.5	2.5	2.9	2.8	0.7	0.5	0.4	0.3
Hazelnuts (g)	1.5	1.5	3.1	3.0	1.2	0.9	--	--
Macadamia Nuts (h)	0.7	0.7	2.1	2.0	0.4	0.3	3.0	2.2
Pistachios (i)	1.5	1.5	3.0	2.9	1.0	0.7	1.0	0.7

RDA (recommended dietary allowance): Zn: 11 mg—men, 8 mg—women; Mg: 420 mg—men, 320 mg—women; Ca: 1000 mg—men and women; Se: 55 µg—men and women. M—male, F—female. INQ—Index of Nutritional Quality; INQ > 1 indicates that a product is a good source of nutrients.

**Table 3:** Bioaccessibility ratios of elements (Zn, Mg, Ca, and Se) (mean value ± SD) from various nuts (Brazil nuts, walnuts, peanuts, almonds, cashew nuts, pecans, hazelnuts, Macadamia nuts, and pistachios) following the in vitro digestion method.

Types of Nuts	Bioaccessibility (%)			
	Zn	Mg	Ca	Se
Brazil Nuts (a)	3.01 ± 1.10	3.45 ± 1.39	0.56 ± 0.41	70.65 ± 23.16
Walnuts (b)	10.73 ± 4.31	6.92 ± 3.15	3.17 ± 0.98	--
Peanuts (c)	5.23 ± 2.38	7.30 ± 2.26	3.25 ± 0.88	34.99 ± 18.63
Almonds (d)	6.43 ± 4.03	6.54 ± 1.98	1.64 ± 0.52	--
Cashew Nuts (e)	5.68 ± 2.17	27.01 ± 5.27	3.14 ± 1.19	--
Pecans (f)	16.96 ± 3.33	10.06 ± 4.66	5.55 ± 2.42	27.72 ± 3.27
Hazelnuts (g)	9.74 ± 7.18	13.04 ± 1.68	2.08 ± 0.61	--
Macadamia Nuts (h)	39.44 ± 7.87	23.92 ± 7.00	8.74 ± 1.77	48.30 ± 16.94
Pistachios (i)	48.10 ± 12.71	15.44 ± 6.58	8.12 ± 3.41	26.81 ± 8.41
Significant differences	*p* < 0.05: a/b, a/c, a/d, a/e, a/f, a/g, a/h, a/i, b/c, b/d, b/e, b/f, b/h, b/i, c/f, c/h, c/i, d/f, d/h, d/i, e/f, e/h, e/i, f/g, f/h, f/i, g/h, g/i.	*p* < 0.05:a/b, a/c, a/d, a/e, a/f, a/g, a/h, a/i, b/e, b/g, b/h, b/i, c/e, c/g, c/h, c/i, d/e, d/f, d/g, d/h, d/i, e/f, e/g, e/i, f/g, f/h, f/i, g/h, h/i.	*p* < 0.05: a/b, a/c, a/d, a/e, a/f, a/g, a/h, a/i, b/d, b/f, b/g, b/h, b/i, c/d, c/f, c/g, c/h, c/i, d/e, d/f, d/h, d/i, e/f, e/g, e/h, e/i, f/g, f/h, g/h, g/i.	*p* < 0.05: a/c, a/f, a/i.

**Table 4:** Correlation between main nut ingredients (energy value, protein, sugars, carbohydrates, fat, and saturated fat) and bioavailability.

Bioaccessibility of Minerals	R Spearman (*p*) Total (n = 90)			
	Protein	Fat	Carbohydrates	Sugars
Zn	−0.29 (*p* < 0.01)	0.23 (*p* < 0.05)	-	0.36 (*p* < 0.001)
Mg	-	-	0.44 (*p* < 0.001)	0.46 (*p* < 0.001)
Ca	-	-	0.35 (*p* < 0.001)	0.40 (*p* < 0.001)

## Data Availability

Data supporting this study are included within the article and supporting materials.

## Supplementary Materials

The following supporting information can be downloaded at: https://www.mdpi.com/article/10.3390/foods12244453/s1, Table S1: Factor loadings for study parameters for the three first principal components.
