# Supplementary material for: In Vitro Assessment of the Bioaccessibility of Zn, Ca, Mg, and Se from Various Types of Nuts
Source: Foods. 2023 Dec 12;12(24):4453. doi: 10.3390/foods12244453 (PMC10742998; doi:10.3390/foods12244453)
Supplement: Supplementary file 1 [file foods-12-04453-s001.zip › foods-2758170-supplementary.pdf]

Table S1. Factor loadings for study parameters for the three first principal components.

Parameters	Loadings		
	PC1	PC2	PC3
Bioavailability of Zn	-0.26	-0.39	0.06
Bioavailability of Mg	-0.22	-0.42	0.19
Bioavailability of Ca	-0.28	-0.41	0.06
Bioavailability of Se	0.30	0.10	0.10
Energy value	0.30	-0.39	-0.02
Fats	0.31	-0.37	-0.06
Saturated fat	0.38	-0.05	0.29
Carbohydrates	-0.37	0.08	0.12
Sugars	-0.39	-0.05	0.40
Fiber	-0.18	-0.17	-0.83
Protein	-0.26	0.40	-0.04
